# Correction: Growth and the pubertal growth spurt in South African adolescents living with perinatally-acquired HIV infection

**DOI:** 10.1371/journal.pone.0306221

**Published:** 2024-06-21

**Authors:** Bilema Mwambenu, Vundli Ramokolo, Ria Laubscher, Ute Feucht

The fourth author’s name is spelled incorrectly. The correct name is: Vundli Ramokolo. The correct citation is: Mwambenu B, Ramokolo V, Laubscher R, Feucht U (2022) Growth and the pubertal growth spurt in South African adolescents living with perinatally-acquired HIV infection. PLOS ONE 17(1): e0262816. https://doi.org/10.1371/journal.pone.0262816.
