# Supplementary material for: Growth and the pubertal growth spurt in South African adolescents living with perinatally-acquired HIV infection
Source: PLoS One. 2022 Jan 25;17(1):e0262816. doi: 10.1371/journal.pone.0262816 (PMC8789101; doi:10.1371/journal.pone.0262816)
Supplement: Attachment — Submitted filename: PLOS ONE Rebuttal Letter 2.12.21 PLOS ONE (1) (1).docx [file pone.0262816.s002.docx]

2 December 2021

To the editor, PLOS ONE

RE: **Growth and the pubertal growth spurt in South African adolescents living with perinatally-acquired HIV infection (PONE-D-21-22182)**

Dear Sir/Madam

Thank you for your valuable time and for considering our manuscript for publication in PLOS ONE. Please find our responses to the reviewers’ comments:

1. **Reviewer 1:** This paper is excellent and bring important data from HIV impacted country. It highlights the result of antiretroviral treatment timing on subsequent delayed growth in adolescent period.

I notice that the results were categorized according to gender of children, and showed consistent pattern in the table and graph. However in the conclusion the author included TB co-infection as one of contributing factor to growth delay. This was not supported by the GLM analysis result, model 2. Should there be other explanation for this, I think the author should revise the conclusion

**Authors’ response**: We thank the reviewer for this comment. We agree that our conclusion does not correlate with the results as TB infection was not significant in the adjusted GLM model 1. We have therefore removed the text relating to TB co-infection in the conclusion.

2. **Reviewer 2:** This is a good information about growth in HIV adolescent with vertical transmission and described the situation in the site. The study reveals 4 times of body measurement and the authors analyzed the Growth velocity graph, is it quite enough? I think it is better make a grouping the patient's age & sex while analyzed the growth velocity instead take all measurement to analysis; because boy and girl have different age of growth velocity.

**Authors’ response**: Thank you for the comment. The growth velocity was calculated and graphs were generated using the mean height change calculated at 6-monthly intervals. We used all data available from the time that ART was initiated, to calculate the annual change. Furthermore, we fully agree that growth in boys and girls differs; therefore, all growth analyses were adjusted for child age and sex by stratification, standardization (the Z-scores) or adjustment in the regression models. The velocity analysis in figure is stratified by sex and takes age into account.

3. Additionally, we identified some errors in table 4, which have been corrected in the updated manuscript, together with a better description of the models in the footnote below the table:

- The chosen reference for age at ART initiation was the group <6 years, instead of the age of ≥6 years
- The asterisks for HIV disease severity and tuberculosis co-infection were incorrect and therefore removed in the table (under model 1)
- More accurate description of the GLM models:
  - Model 1 is a descriptive model including all variables and assessing HAZ at study exit
  - Model 2 is a predictive model and only included variables significantly associated with HAZ at study exit.

4. We also adjusted by 4, the numbers of participants that were excluded from our study, as shown in the text, as it had been an oversight to not deduct them from the total number of participants in the initial description of the cohort.

Thank you once again for the valuable feedback, and we look forward to hearing from you.

Dr Mwambenu
